# Stercoral ulcer after Hirschsprung's disease surgery

**DOI:** 10.1002/ccr3.5239

**Published:** 2021-12-26

**Authors:** Toshihiko Kakiuchi, Atsuhisa Fukuta, Koichiro Yoshimaru, Yumeng Zhang, Ryo Shimoda, Muneaki Matsuo

**Affiliations:** ^1^ Department of Pediatrics, Faculty of Medicine Saga University Saga Japan; ^2^ Department of Pediatric Surgery Kyushu University Hospital Fukuoka Japan; ^3^ Division of Gastroenterology, Department of Internal Medicine, Faculty of Medicine Saga University Saga Japan

**Keywords:** constipation, Down syndrome, Hirschsprung's disease, pressure ulcer

## Abstract

Constipation and stercoral ulcer are risk factors associated with Hirschsprung's disease (HD); long‐term follow‐up is, thus, essential. In postoperative HD associated with Down syndrome (DS) with intellectual disability, vigilant follow‐up is required to avoid severe constipation because DS predisposes the patients to constipation and caregivers cannot easily understand the symptoms.

## Graphical abstract

Patients with postoperative Hirschsprung's disease associated with Down syndrome with intellectual disability require careful and long‐term follow‐up to avoid severe constipation that may lead to stercoral ulcer.

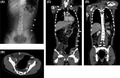

## 1 INTRODUCTION

Stercoral ulcer is a pressure ulcer due to impaired blood flow resulting from severe constipation where stagnant stool compresses the large intestinal wall.^1^ This type of ulcer is usually observed in the cases of severe constipation in older individuals with underlying illness and who are bedridden; however, it is rarely observed in younger individuals.^2^ Stercoral ulcer occurs when chronic constipation leads to fecal impaction and fecal stone development, which are the masses of dehydrated fecal matter. The fecalith remains within the area of the colon, primarily in the rectal sigmoid colon, leading to the area of ischemic pressure ulcers, eventually causing localized ischemic necrosis and colon perforation.^3^

Hirschsprung's disease (HD) is a congenital disorder characterized by the lack of ganglion cells in the submucosal and myenteric plexuses and the lack of peristalsis in the intestinal tract, causing functional intestinal obstruction. It is surgically treated by pulling down an intestinal section with normal innervation to the anus where the anal sphincter is preserved. This HD procedure generally has no complications. However, up to 10% and less than 1% of patients after HD surgery may suffer from constipation and fecal incontinence, respectively.^4^

Here, we describe a case of severe anemia caused by stercoral ulcer in a young patient with Down syndrome (DS) after HD surgery.

## 2 CASE PRESENTATION

**FIGURE 1:**
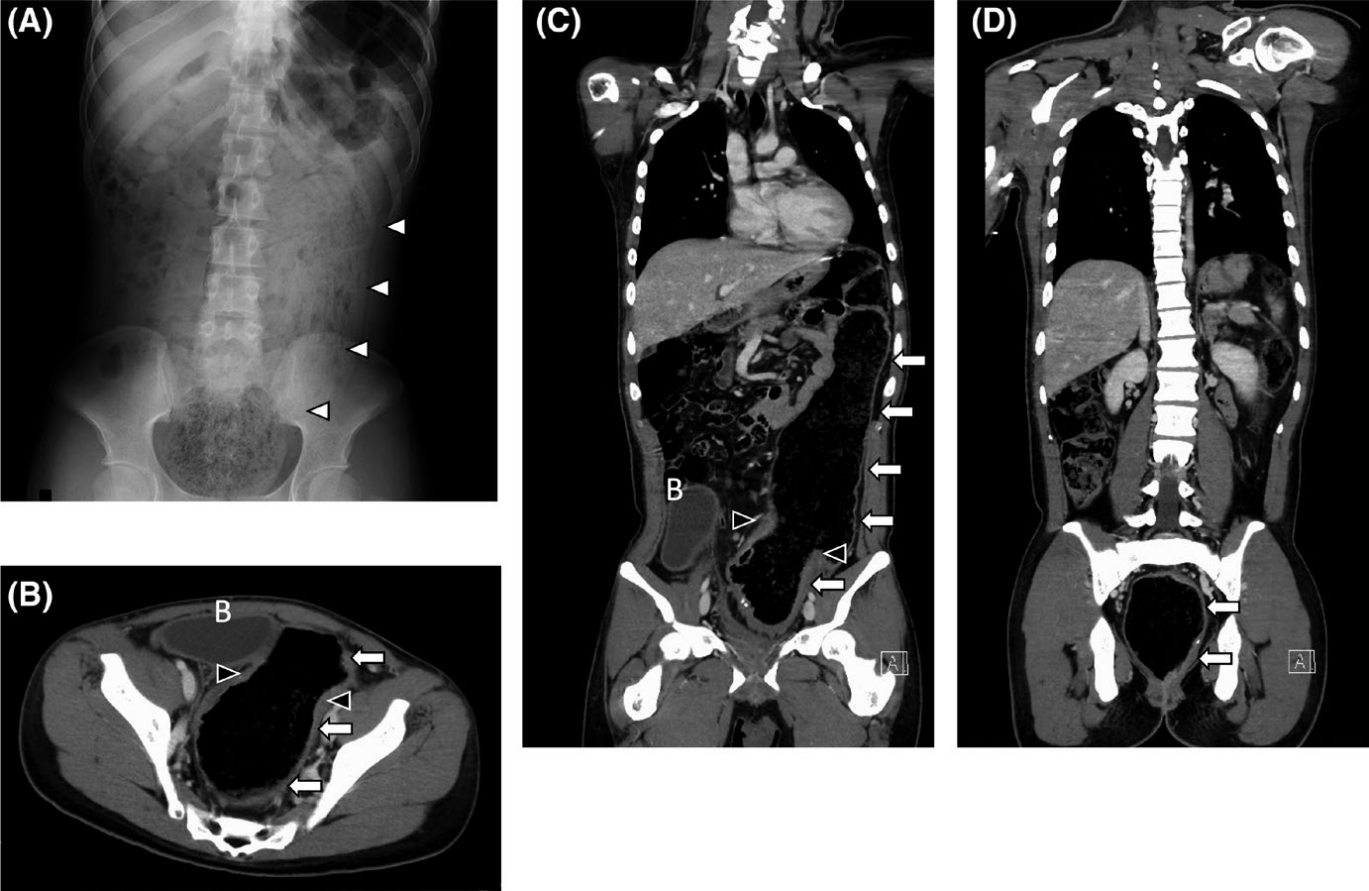
(A) Finding of abdominal X‐ray showed a stool mass from the descending colon to the rectum. Axial (B) and coronal (C, D) dislocation of abdominal enhanced computed tomography showed a highly dilated large intestine, large amounts of residue, and gas retention. White arrows; dilated intestinal tract, white arrowheads; stool mass, black arrowheads; anastomotic site, B; bladder

**FIGURE 2:**
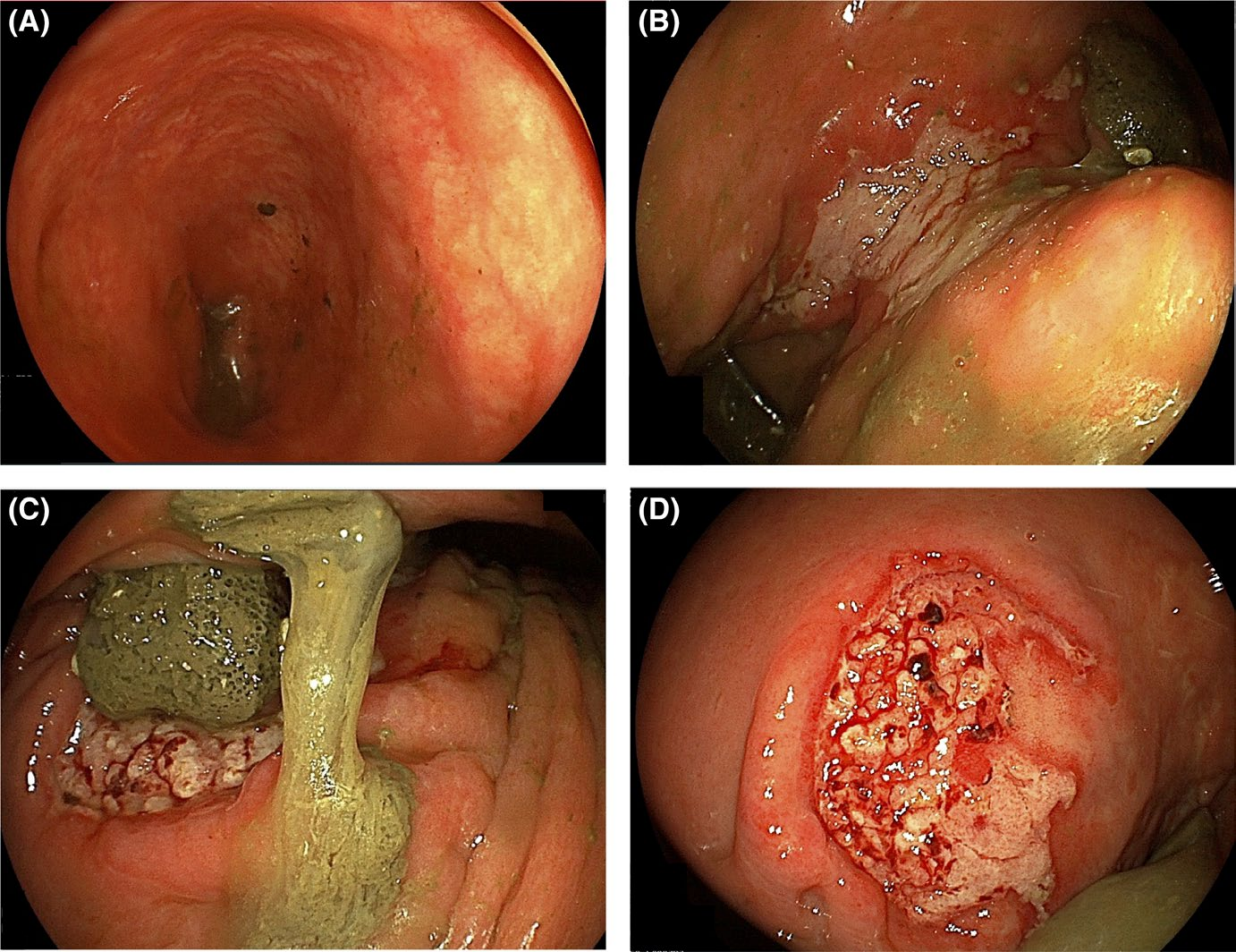
Findings of total colonoscopy were that a shallow ulcer was found at the anastomotic site (B), and a shallow irregular ulcer with oozing on the anal side (C, D). Hard stool mass was attached to the ulcer site (C). Intestinal tract on the oral side of the anastomosis was slightly edematous, but no obvious ulcer or bleeding was observed (A)

**FIGURE 3:**
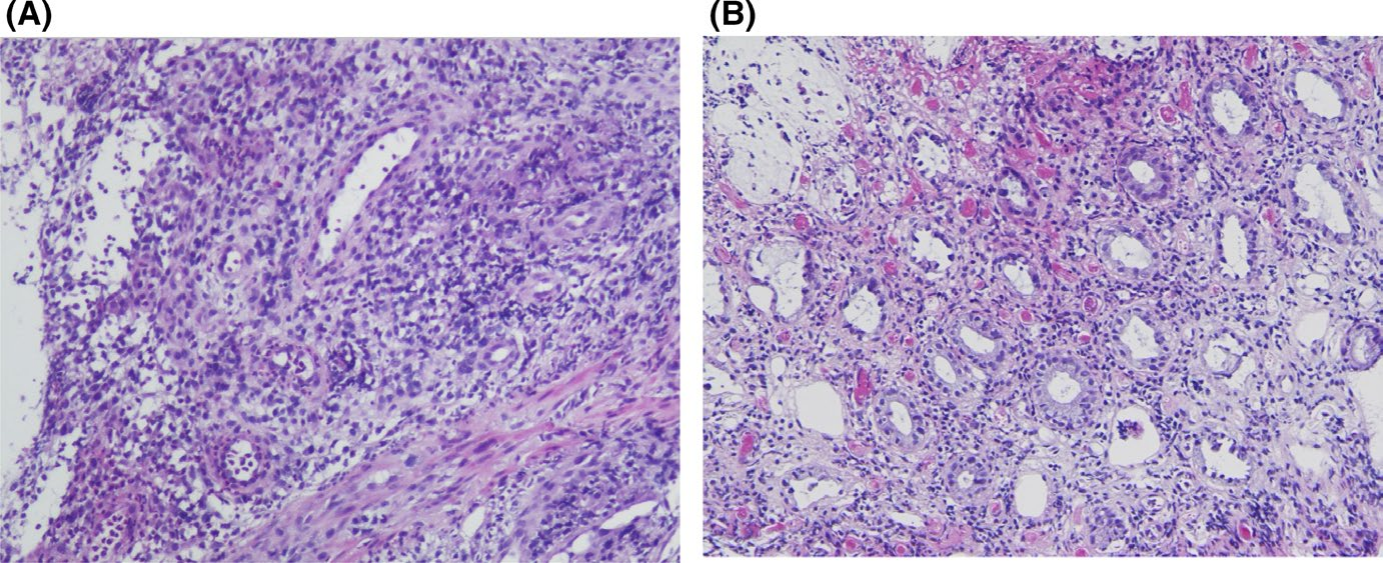
Pathological finding showed that the columnar epithelium was shed in the superficial part of the mucosa, and the glandular duct was atrophied (A). Large number of fibrin thrombi in small blood vessels appeared; it was a finding consistent with ischemic changes (B)

A 19‐year‐old man with DS was admitted to our hospital with severe anemia (hemoglobin, 5.8 g/dl) and 3‐month passage of black stool. Shortly after birth, he was diagnosed with HD from delayed meconium excretion and abdominal distension, and underwent stoma construction, followed by Duhamel surgery and Z‐type anastomosis at 2 months of age. The patient had no postoperative complications, with almost no hospital follow‐up for the last 19 years, as reported by the guardian. In addition, his parents claimed no gastrointestinal symptoms during the 19 years. He seemed to defecate once every 2 days, with a normal stool form (Bristol stool scale: 4–5), but the fact remained uncertain. He had DS with intellectual disability and had difficulty communicating; thus, physicians could hardly grasp his symptoms. His lab data showed no abnormalities other than anemia, and thyroid function and urinalysis were normal. Abdominal X‐ray detected a stool‐like mass from the descending colon to the rectum (Figure 1A). In enhanced computed tomography (CT), a highly dilated large intestine, large residues, and gas retention were observed (Figure 1B,C,D). The bladder was squeezed into the dilated descending colon and was largely displaced to the right side. Esophagogastroduodenoscopy demonstrated no abnormal findings. However, total colonoscopy revealed a shallow ulcer at the anastomotic site (Figure 2B) and a shallow irregular ulcer with oozing at the anal side (Figure 2C,D). A hard stool mass was attached to the ulcer site (Figure 2C). The intestinal tract on the anal side of the anastomosis was slightly edematous, with no apparent ulcer or bleeding (Figure 2A). In the gastrointestinal mucosal pathology of the ulcer, the columnar epithelium was shed in the superficial part of the mucosa, with glandular duct atrophy (Figure 3A). Additionally, numerous fibrin thrombi were detected in small blood vessels, suggesting ischemic changes (Figure 3B). Hence, the diagnosis was stercoral ulcer. The cause of his anemia was diagnosed as gastrointestinal bleeding from stercoral ulcer. Urgent red blood cell transfusion was performed to improve severe anemia. Considering the difficulty of communicating with the patient with DS, enema treatment was challenging. In fact, he did not cooperate and stubbornly refused regular enema treatment. Thus, several laxatives were administered, and defecation was controlled twice daily. Eventually, hematochezia and anemia disappeared as defecation was well‐controlled.

## 3 DISCUSSION

The present case suggests that defecation must be strictly managed to avoid severe constipation, which may cause stercoral ulcer, post‐HD surgery.

In the present case, various factors contributed to severe constipation after HD surgery. Through digital rectal examination, anal obstruction was ruled out. Rectal dilatation findings in CT revealed no anastomotic stenosis. Given that bloody stools and defecation were controlled with laxatives after the diagnosis, owing to the long aganglionic segment in the pull‐through intestinal tract, it is unlikely to have caused severe constipation. This could be observed from the fact that our patient did not suffer from persistent constipation for a few years after HD surgery. In our patient's case, constipation worsened at the time of visiting our facility, expanding the colon enormously. Constipation seemingly worsened when defecation was improperly managed because of difficulty in communication and the lack of medical follow‐up for a long period of time at the discretion of the parents. Due to worsening constipation, stercoral ulcer causing bloody stools and severe anemia is considered to have occurred.

Stercoral ulcer often occurs in patients with a medical history of chronic constipation, older individuals with dementia, bed‐bound patients, and sometimes young patients with mental illness.^5^ Chronic constipation is the main cause for developing stercoral ulcer.^2^ Stercoral ulcer can be easily diagnosed if the ulcer is found on the stagnation site of feces. The most feared complications associated with stercoral ulcer are gastrointestinal perforation and ischemic colitis. Because it poses a risk of bloody stools and gastrointestinal perforation, stercoral ulcer has a poor prognosis.^6^, ^7^

Down syndrome is a common human chromosomal disorder. Among the clinical findings, the high prevalence of gastrointestinal system alterations is a constant concern.^8^ In patients with DS, gastrointestinal disorders are the most common anomalies and have a significant impact on their daily life,^9^with chronic intestinal constipation as the most prevalent.^8^, ^10^Patients with DS often have difficulty communicating properly with others, thereby leading to inadequate provision of daily health care and the oversight of serious illnesses.

The clinical association between DS and HD is well‐established. Approximately 5% of HD cases are associated with DS, and as a result, DS remains the most common congenital disorder associated with HD.^11^, ^12^ Considering that up to 10% of patients with HD may have constipation after HD surgery^4^ and that DS often exhibits chronic intestinal constipation,^8^, ^10^ patients with DS require strict defecation control after undergoing HD surgery.

Risto et al. reported that the overall incidence of constipation with post‐HD surgery might increase with increasing age; for instance, constipation occurred in 30% of patients after undergoing Duhamel procedure and 10% of them treated their constipation with laxatives or enemas.^13^, ^14^ Menezes et al. reported that on comparing the patients with DS after HD surgery with those of non‐DS after HD surgery, there were high incidences in the rates of soiling and continence in the patients with DS. Similar to the present case, patients with DS after the Duhamel procedure for HD need to be particularly careful, even in the future, of the detrimental sequelae of constipation. In the present case, the patient developed stercoral ulcer 19 years after HD surgery.

In conclusion, patients with postoperative HD associated with DS with intellectual disability require careful and long‐term follow‐up to avoid severe constipation that may lead to stercoral ulcer.

## CONFLICT OF INTEREST

The authors declare that the study was conducted in the absence of any commercial or financial relationships that could be construed as a potential conflict of interest. There was no external funding in the preparation of this manuscript.

## AUTHOR CONTRIBUTIONS

Dr. Kakiuchi, Dr. Fukuta, Dr. Yoshimaru, and Dr. Zhang were involved in patient care as well as the drafting, review, and revision of the initial manuscript. Dr. Shimoda and Dr. Matsuo were involved in patient care and project administration as well as the review and revision of the initial manuscript. All authors have approved the final manuscript and agree to be accountable for all aspects of the study.

## ETHICAL APPROVAL

Ethical review and approval of the study on human participants in accordance with the local legislation and institutional requirements were not required in this case.

## CONSENT

Written informed consent to publish this case was provided by the patient and his family.

## Data Availability

The datasets used and/or analyzed during the present study are available from the corresponding author on reasonable request.

## References

[1] Gough AE , Donovan MN , Grotts J , Greaney GC . Perforated stercoral ulcer: a 10‐year experience. J Am Geriatr Soc. 2016;64:912‐914. 27100603 10.1111/jgs.14057

[2] Heffernan C , Pachter HL , Megibow AJ , Macari M . Stercoral colitis leading to fatal peritonitis: CT findings. AJR Am J Roentgenol. 2005;184:1189‐1193. 15788592 10.2214/ajr.184.4.01841189

[3] Naseer M , Gandhi J , Chams N , Kulairi Z . Stercoral colitis complicated with ischemic colitis: a double‐edge sword. BMC Gastroenterol. 2017;17:129. 29179680 10.1186/s12876-017-0686-6 PMC5704496

[4] Coran AG , Teitelbaum DH . Recent advances in the management of Hirschsprung's disease. Am J Surg. 2000;180:382‐387. 11137692 10.1016/s0002-9610(00)00487-6

[5] Ünal E , Onur MR , Balcı S , Görmez A , Akpınar E , Böge M . Stercoral colitis: diagnostic value of CT findings. Diagn Interv Radiol. 2017;23:5‐9. 27910814 10.5152/dir.2016.16002 PMC5214077

[6] Vijayakumar C , Balagurunathan K , Prabhu R , et al. Stercoral ulcer not always indolent: a rare complication of fecal impaction. Cureus. 2018;10:e2613. 30027005 10.7759/cureus.2613 PMC6044477

[7] Marget M , Ammar H . Not your usual constipation: stercoral perforation. BMJ Case Rep. 2017;2017;bcr2016218283. 10.1136/bcr-2016-218283 PMC5318602 28193645

[8] Bermudez B , de Oliveira CM , de Lima Cat MN , Magdalena NIR , Celli A . Gastrointestinal disorders in down syndrome. Am J Med Genet A. 2019;179:1426‐1431. 31183986 10.1002/ajmg.a.61258

[9] Ravel A , Mircher C , Rebillat AS , Cieuta‐Walti C , Megarbane A . Feeding problems and gastrointestinal diseases in down syndrome. Arch Pediatr. 2020;27:53‐60. 31784293 10.1016/j.arcped.2019.11.008

[10] Wallace RA . Clinical audit of gastrointestinal conditions occurring among adults with down syndrome attending a specialist clinic. J Intellect Dev Disabil. 2007;32:45‐50. 17365367 10.1080/13668250601146761

[11] Moore SW . Advances in understanding the association between down syndrome and Hirschsprung disease (DS‐HSCR). Pediatr Surg Int. 2018;34:1127‐1137. 30218169 10.1007/s00383-018-4344-z

[12] Ieiri S , Higashi M , Teshiba R , et al. Clinical features of Hirschsprung's disease associated with down syndrome: a 30‐year retrospective nationwide survey in Japan. J Pediatr Surg. 2009;44:2347‐2351. 20006024 10.1016/j.jpedsurg.2009.07.055

[13] Rintala RJ , Pakarinen MP . Long‐term outcomes of Hirschsprung's disease. Semin Pediatr Surg. 2012;21:336‐343. 22985839 10.1053/j.sempedsurg.2012.07.008

[14] Jarvi K , Laitakari EM , Koivusalo A , Rintala RJ , Pakarinen MP . Bowel function and gastrointestinal quality of life among adults operated for Hirschsprung disease during childhood: a population‐based study. Ann Surg. 2010;252:977‐981. 21107107 10.1097/SLA.0b013e3182018542

